# An interventional quasi-experimental study to evaluate the impact of a rapid screening strategy in improving control of nosocomial extended-spectrum beta-lactamase-producing Enterobacterales and carbapenemase-producing organisms in critically ill patients

**DOI:** 10.1186/s13054-022-04027-8

**Published:** 2022-06-07

**Authors:** Romain Martischang, Patrice François, Abdessalam Cherkaoui, Gesuele Renzi, Carolina Fankhauser, Jacques Schrenzel, Jérôme Pugin, Stephan Harbarth

**Affiliations:** 1 Infection Control Program, Geneva University Hospitals, Geneva, Switzerland; 2 Genomic Research Laboratory, Geneva University Hospitals, Geneva, Switzerland; 3 Bacteriology Laboratory, Geneva University Hospitals, Geneva, Switzerland; 4 Intensive Care Units, Geneva University Hospitals, Geneva, Switzerland

**Keywords:** Screening, Surveillance, Infection control, Contact precautions, Multiresistant Gram-negative bacteria, ICU, Clinical study, Molecular test

## Abstract

**Introduction:**

Rapid molecular tests could accelerate the control of extended-spectrum beta-lactamase-producing Enterobacterales (ESBL-PE) and carbapenemase-producing organisms (CPO) in intensive care units (ICUs).

**Objective and methods:**

This interventional 12-month cohort study compared a loop-mediated isothermal amplification (LAMP) assay performed directly on rectal swabs with culturing methods (control period, 6 months), during routine ICU screening. Contact precautions (CP) were implemented for CPO or non-*E. coli* ESBL-producing Enterobacterales (nEcESBL-PE) carriers. Using survival analysis, we compared the time intervals from admission to discontinuation of unnecessary preemptive CP among patients at-risk and the time intervals from screening to implementation of CP among newly identified carriers. We also compared diagnostic performances, and nEcESBL-PE/CPO acquisition rates. This study is registered, ISRCTN 23588440.

**Results:**

We included 1043 patients. During the intervention and control phases, 92/147 (62.6%) and 47/86 (54.7%) of patients at-risk screened at admission were candidates for early discontinuation of preemptive CP. The LAMP assay had a positive predictive value (PPV) of 44.0% and a negative predictive value (NPV) of 99.9% for CPO, and 55.6% PPV and 98.2% NPV for nEcESBL-PE. Due to result notification and interpretation challenges, the median time from admission to discontinuation of preemptive CP increased during the interventional period from 80.5 (95% CI 71.5–132.1) to 88.3 (95% CI 57.7–103.7) hours (*p* = 0.47). Due to the poor PPV, we had to stop using the LAMP assay to implement CP. No difference was observed regarding the incidence of nEcESBL-PE and CPO acquisition.

**Conclusion:**

A rapid screening strategy with LAMP assays performed directly on rectal swabs had no benefit for infection control in a low-endemicity setting.

**Supplementary Information:**

The online version contains supplementary material available at 10.1186/s13054-022-04027-8.

## Introduction

Digestive carriage of extended-spectrum beta-lactamase-producing Enterobacterales (ESBL-PE) and carbapenemase-producing organisms (CPO) places patients at-risk of antibiotic-resistant infection, increasing length of hospital stay [1, 2] and mortality [2, 3]. Active surveillance as part of a multimodal approach already proved to be efficient to decrease ESBL and CPO infections [4, 5]. In intensive care units (ICUs), admission and weekly universal screenings help to detect new CPO and ESBL-PE carriers. Among patients at-risk, preemptive contact precautions (CP) after admission may be discontinued after negative results. However, current microbiologic screening methods are slow, delaying the discontinuation of preemptive CP, with possible harmful effects [6, 7]. This diagnostic delay also impacts detection of previously unknown carriers screened during routine surveillance, leading to an increased risk of cross-transmission.

Molecular screening methods such as loop-mediated isothermal amplification (LAMP) reaction assays have been developed to improve diagnostic performance [8] and accelerate the slow turn-around times observed with traditional culture-based systems [9–11]. However, several reviews recently stressed the lack of clinical effectiveness studies [12, 13]. In order to improve CPO and ESBL-PE control in the ICU setting, LAMP assays may yet represent a reasonably fast and specific, but also cost-effective screening method [14]. We hypothesized that a rapid LAMP assay performed directly on rectal swabs could yield individual and ecological benefits as compared to traditional phenotypic methods, by accelerating the discontinuation of unnecessary preemptive CP for negative patients screened at admission, and by faster implementation of CP for newly identified carriers, thus reducing ESBL-PE and CPO incidence among critically ill patients.

## Material and methods

### Setting and population

Geneva University Hospitals is a tertiary care center with a mixed medical–surgical adult ICU of 30 beds (1/3 single-bed rooms and 2/3 shared rooms), with up to 18 extra-architectural beds used during the COVID-19 pandemic. This ICU admits 2500 patients per year with a median length of stay of 1.9 days. The mean weekly prevalence of ESBL-PE carriage was 10.2% in 2016, with an average of 2.4 newly identified ESBL-PE positive patients per week.

All ICU patients with a surveillance screening for ESBL-PE or CPO by rectal swabs or stool cultures were included in this study. The impact of rapid screening tests on de-implementation of preemptive CP was evaluated on a first sub-group of patients at-risk of ESBL-PE and CPO carriage, screened at admission. The impact on implementation of targeted CP was further evaluated on a second sub-group of patients screened weekly during ICU stay. Patients with a competing and microbiologically proven indication for CP were excluded.

### Study design

This prospective, interventional, quasi-experimental, de-adoption study compared a rapid molecular test (LAMP assay) performed directly on all rectal swabs during a first 12-month intervention period (April 2019–March 2020) against conventional culturing methods during a second 6-month control period (May–October 2020), after a 1-month wash-out period.

### Outcomes and definitions

The primary outcome was the median time interval from admission to discontinuation of unnecessary preemptive CP among patients at-risk screened upon ICU admission. Secondary outcomes included the median time from screening to implementation of CP among newly identified carriers, laboratory turn-around-times (TATs), diagnostic performances, and ICU-acquired non-*E. coli* ESBL-producing Enterobacterales (nEcESBL-PE) or CPO acquisition events, defined as a newly detected nEcESBL-PE or CPO carriers by screening or clinical culture. Incidence rates of nEcESBL-PE and CPO acquisition were defined per 1000 patient-days at-risk.

### Surveillance screening and infection control measures

Admission screening targeted patients with specific risk profiles (patients at-risk) (Table 1). We distinguished patients at higher risk profile (patients at high risk) requiring confirmatory screening. Weekly universal screening was performed for all ICU patients present on Monday morning. Additional screening of roommates was performed during active case finding in case of cluster investigations.

**Table 1:** Exposures considered for admission screening

*Indication for ESBL-PE and CPO screening at admission of patients at-risk*
Hospitalized and overnight stay in Switzerland
Prior travel in endemic countries
Dialysis treatment
*Indication for ESBL-PE and CPO screening at admission of high risk patients*
Known ESBL-PE and CPE carrier
Hospitalized and overnight stay abroad

nEcESBL-PE or CPO carriers were identified by door signage, flagged using automatized alert systems, and placed under CP, which included dedicated material (gowns, gloves), spatial separation, and environmental decontamination. Preemptive CP were discontinued at the first negative result for patients at-risk, or after sequential screenings for patients at high risk (e.g., previously known CPO carrier). Microbiological results were actively screened by dedicated infection control nurses, to ensure adequate discontinuation or implementation of infection control measures. Timing and adequacy of prescription for screening and CP were monitored by a dedicated nurse. Environmental cleaning was temporarily modified during a *P. aeruginosa* outbreak, focusing on controlling the aquatic reservoir (disinfection and suppression of ICU sinks in September 2020) [15].

### Microbiological procedures

#### Routine screening procedures with conventional culture methods

Rectal swabs (eSwab™, Copan) were routinely collected by trained ICU nurses. Swabs were then plated on three media: chromID ESBL (BioMérieux), chromID® OXA-48 (BioMérieux), and CHROMagar™ *Acinetobacter* (CHROMagar, France). All colonies with specific colors defined by the manufacturers’ instructions were identified by matrix-assisted laser desorption ionization–time of flight (MALDI-TOF) mass spectrometry, and the antibiotic susceptibility profile of each isolate was determined by the disk diffusion method using EUCAST recommendations [16]. For ESBL confirmation, we used double-disk synergy tests. In doubtful cases, ESBL + AmpC Screen Kit 98008 (Rosco Diagnostica) were used as a second line confirmatory test. For CPO confirmation, we used the LAMP eazyplex® SuperBug CRE system (AxonLab, UK) on selected isolates, a qualitative molecular test covering CTX M-1 and CTX M-9 families, KPC variants (KPC2 to KPC15), NDM variants (NDM1 to NDM7), VIM variants (VIM1 to VIM37), OXA-48-like variants (OXA-48, OXA-162, OXA-204, and OXA-244), and OXA-181-like variants (OXA-181, and OXA-232).

#### Workflow

The bacteriology laboratory processed nonstop all diagnostic samples related to the study during weekdays until 17h00. Of note, plating of isolates, incubation, and culture triage were automatized from March 2019 onward [17].

#### Interventional screening strategy (LAMP assay)

Rectal swabs were split into three equal parts and processed simultaneously. A first part was run by LAMP eazyplex® SuperBug CRE system as described above to detect the main genes encoding for ESBLs and carbapenemases. A second part was processed using standard bacteriology methods for pathogen identification and quality assurance purposes (i.e., to confirm the presence of *E. coli*). A third part was stored at − 20 °C to resolve any potential discordance between molecular and phenotypic results. The results were communicated in real time from Monday to Friday.

Unnecessary preemptive CP were stopped based on negative LAMP results, and CP were implemented for newly identified patients based on LAMP-positive results for CPO, or culture-positive results for nEcESBL-PE. Cultures were used as the reference test in case of discordant results. LAMP-positive and culture-negative samples were investigated post hoc using specific PCRs (TEM, SHV). Isolates from samples negative by LAMP were retested using LAMP and disk diffusion methods.

#### Pilot test

This rapid testing strategy has been previously validated in our institution and showed high sensitivity and specificity [18]. In 2018, we included 209 samples from 187 ICU patients and observed a TAT gain of 44.1 hours with the LAMP technology compared to conventional methods, with a high specificity and negative predictive value (98.8% and 97.6%, respectively) [19]. In the present clinical study, the diagnostic performance of LAMP was again evaluated among all samples processed by both LAMP and cultures, the latter being used as a reference test.

### Data collection

TATs were categorized into pre-analytical TAT (time from admission to screening, time from screening to sample delivery to the laboratory), analytical TAT (time from arrival at the laboratory to reporting of results), and post-analytical TAT (time from result notification to implementation or discontinuation of CP). Pre-analytical and analytical TATs were collected from computerized laboratory databases. Post-analytical TAT was computed based on the date and time of implementation or discontinuation of CP, directly informed by the electronic patient file.

Acquisition events were collected using screening and clinical cultures from routine surveillance data. Colonization pressure was defined as the monthly sum of positive screening and clinical cultures for ESBL-PE and CPO. Only the first ESBL-PE or CPO isolate was considered per patient. Monthly hand hygiene compliance of healthcare workers was collected according to WHO methods. Systemic antibiotic consumption was measured in daily doses per 1000 patient-days for all antibiotics (ATC J01), and for antibiotics of interest (beta-lactams, carbapenems, aminoglycosides, polymyxin). Adherence to screening and contact precautions was measured as defined in Additional file 1: Appendix S1.

### Statistical analysis

#### Time benefits for infection control

Analytical TATs expressed as medians were first compared using Wilcoxon rank-sum test, *χ*^2^ test, and Fisher exact test when appropriate. Unnecessary times (in days) spent under preemptive CP among patients screened at admission were compared for the intervention and control periods using survival analysis. Right censoring of patients occurred at ICU discharge or death, which were consequently regarded as competing events. Proportional subdistribution hazard modeling was performed in addition to cause-specific hazard models to account for competing events. Subdistribution hazard ratio (sHR) and cause-specific hazard ratios (csHR) were calculated using Fine and Grey models, and Cox models, respectively. The proportional hazards assumption was tested by the visual examination of Schoenfeld residuals.

TATs were evaluated in an intention-to-treat analysis, regardless of the patient status (at-risk or at a high risk) and study-related laboratory activity, which was interrupted during weekends and public holidays. Several exploratory analyses were also performed. First, to estimate the effect of the rapid screening strategy on actionable results (without the need of sequential screening), we performed the same analysis, excluding patients screened during holidays and at high risk. Second, we reported detailed pre-analytical, analytical, and post-analytical TATs of patients screened at admission with CP discontinuation before discharge, excluding patients screened during holidays and at high risk.

#### Impact on nEcESBL-PE and CPO acquisition rates

χ2 or Fisher’s exact tests were used to compare categorical variables and Student’s t-test, for continuous variables. The impact of the interventional screening strategy on adjusted incidence density ratios of nEcESBL-PE or CPO acquisition was evaluated using Poisson regression, accounting for aggregate-level exposures, including colonization pressure and hand hygiene compliance. All analyses were performed using R (version 4.0).

### Ethical statement

This study was approved by the local Ethics Committee Review. It was considered as a quality improvement project, relying on routine surveillance data, and was therefore exempted from individual patient consent. This study is registered, ISRCTN 23588440.

## Results

**Fig. 1:**
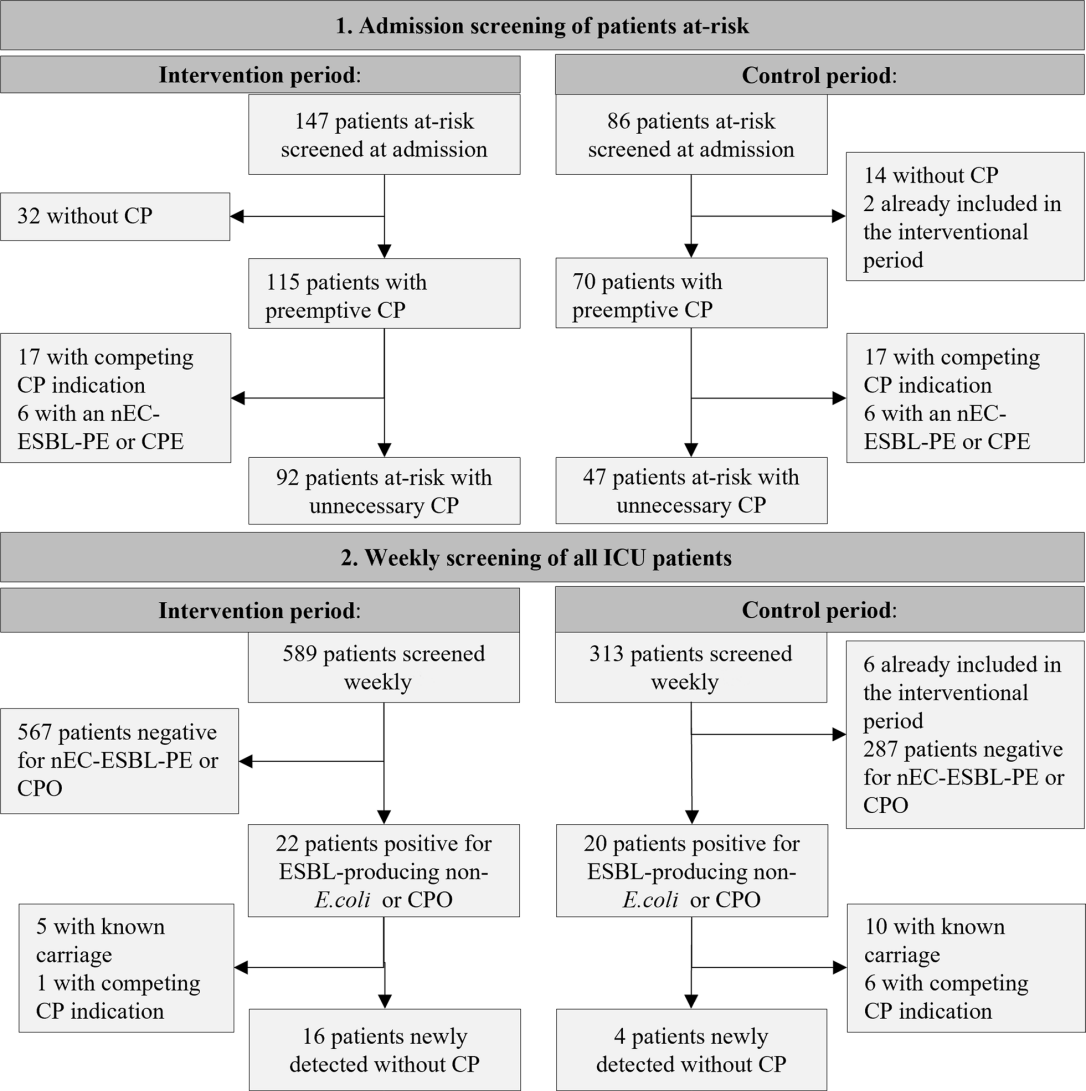
Flowchart of included ICU patients

This study included 1043 patients (median length of stay, 2.2 days) sampled 1778 times, including 231 patients with a targeted screening at admission and 896 patients with either weekly or epidemiologically indicated screening (Fig. 1). Of 231 patients screened at admission, we distinguished 58 (25.1%) patients at high risk requiring sequential screening, and 173 (75.0%) patients at-risk (Table 2). Most patients at-risk were transferred from another hospital (53.2%), and most patients at a high risk were already known carriers (46.6%, Additional file 1: Appendix S2). Among all patients screened at admission, 185 (80.1%) had preemptive CP. Among them¸ only 12 (6.5%) were positive for nEcESBL-PE or CPO, and 34 (18.4%) had an alternative indication to maintain CP, including known carriage of other MDROs. Thus, 139 (75.1%) patients screened at admission were included in the analysis (Fig. 1).

**Table 2:** Individual and aggregated characteristics of study participants and ICU patients

Among participants screened at admissions	Interventional period	Control period	*p* value
	*n* = 147 patients^a^	*N* = 86 patients^a^	
Patients at a high risk (%)	30 (20.4%)	29 (33.7%)	0.04
Median length of stay (days, IQR)	2.8 (1.5–5.6)	2.8 (1.7–7.1)	0.67
nEcESBL-PE carriers	10 (6.8%)	8 (9.3%)	0.68
CPO carriers	1 (0.7%)	3 (3.5%)	0.14
CPE carriers	1 (0.7%)	3 (3.5%)	0.14
Preemptive contact precautions prescribed	115 (78.2%)	70 (81.4%)	0.45

Among all participants screened weekly	*n* = 589 patients^b^	*n* = 313 patients^b^	*p* value
Median length of stay (days, IQR)^c^	6.9 (2.9–12.6)	6.9 (3.8–15.1)	0.06
nEcESBL-PE carriers	19 (3.2%)	16 (5.1%)	0.22
CPO carriers	4 (0.7%)	8 (2.6%)	0.03
CPE carriers	3 (0.5%)	3 (1.0%)	0.42
Newly detected carriers	13 (2.2%)	10 (3.2%)	0.52
Contact precautions implemented at the time of detection among newly detected carriers	1/13 (7.7%)	6/10 (60%)	0.02

Colonization pressure (incidence density)	*n* = 8884 patient-days	*n* = 3772 patients-days	*p* value
ESBL-PE (cases per 1000 patient-days)	15.3 [95% CI 12.8–18.1]	19.1 [95% CI 14.9–24.0]	0.13
CPO (cases per 1000 patient-days)	0.7 [95% CI 0.2–1.5]	2.9 [95% CI 1.5–5.2]	0.05
CPE (cases per 1000 patient-days)	0.4 [95% CI 0.1–1.1]	1.6 [95% CI 0.6–3.5]	0.22

Antibiotic consumption (ATC JC01)	*n* = 8884 patient-days	*n* = 3772 patients-days	*p* value
Aminoglycoside (median DDD/1000 patient-days)	19.2 [95% CI 8.2–26.8]	22.5 [95% CI 18.3–29.5]	0.44
Carbapenems (median DDD/1000 patient-days)	97.0 [95% CI 66.9–129.0]	126.0 [95% CI 104.0–133.0]	0.18
Piperacillin and beta-lactamase inhibitor (median DDD/1000 patient-days)	145.0 [95% CI 128.0–168.0]	114.0 [95% CI 96.1–128.0]	0.03

Hand hygiene compliance among healthcare workers	Hand hygiene opportunities = 474	Hand hygiene opportunities = 360	*p* value
Pooled mean of hand hygiene compliance	59.7 [95% CI 55.3–64.1]	61.1 [95% CI 56.1–66.1]	0.73

^a^Two patients screened at admission were readmitted during the control phase.

^b^Six patients stayed both in the interventional and control phase.

^c^Information available for 151 and 312 patients in the interventional and control period.

As shown in Fig. 1, 42 (4.7%) patients screened routinely during ICU stay were positive by either LAMP or culture for either CPO or nEcESBL-PE. After exclusion of 22 patients with known carriage or competing indication for CP, 20 patients were included in the analysis.

Colonization pressure was similar between interventional and control periods for both ESBL-PE and CPE, but not for CPO, which increased during the control period (Table 2). Median antibiotic consumption and hand hygiene compliance were also similar between both periods, though minor monthly variations occurred (Table 2, Additional file 1: Table S1, Figure S1). However, we observed that consumption of piperacillin–tazobactam slightly increased during the interventional period, potentially influenced by the first COVID-19 pandemic wave.

Adherence to weekly screening during both interventional and control phases was 69.6% [95% CI 61.5–77.5] and 84.0% [95% CI 71.1–87.8], respectively. Lowest screening rates were observed in March 2020 at the peak of the first pandemic wave (Additional file 1: Figure S2). We performed 23 audits to assess implementation of CP. An agreement of 94.0% (146/156 observations) was observed between prescribed and implemented CP.

Among all screened patients, ESBL-PE prevalence was 16.1% (168/1043), including 4.3% (45/1043) of nEcESBL-PE and 1.3% (14/1043) of CPO. Most of the nEcESBL-PE and CPO were isolated in patients at high risk (respectively, 16% and 5%; Additional file 1: Table S2).

Among 1117 samples, including 25 samples CPO-positive by LAMP, the diagnostic performance indicators were: 91.7% [95% CI 76.0–100.0] sensitivity, 98.7% [95% CI 98.1–99.4] specificity, 44.0% [95% CI 24.5–63.5] positive predictive value (PPV), and 99.9% [95% CI 99.7–100.0] negative predictive value (NPV). Among 27 samples nEcESBL-PE positive by LAMP, performances were: 45.4% [95% CI 28.5–62.4] sensitivity, 98.8% [95% CI 98.1–99.5] specificity, 55.6% [95% CI 36.8–74.3] PPV, and 98.2% [95% CI 97.3–99.0] NPV. To note, specificity and NPV observed among CPO (98.7%, 100.0%) and nEcESBL-PE (96.0%, 92.0%) decreased among patients at a high risk screened at admission. Further analysis of discordant results unveiled that most of isolates negative by LAMP were positive once retested (Additional file 1: Appendix S3).

**Fig. 2:**
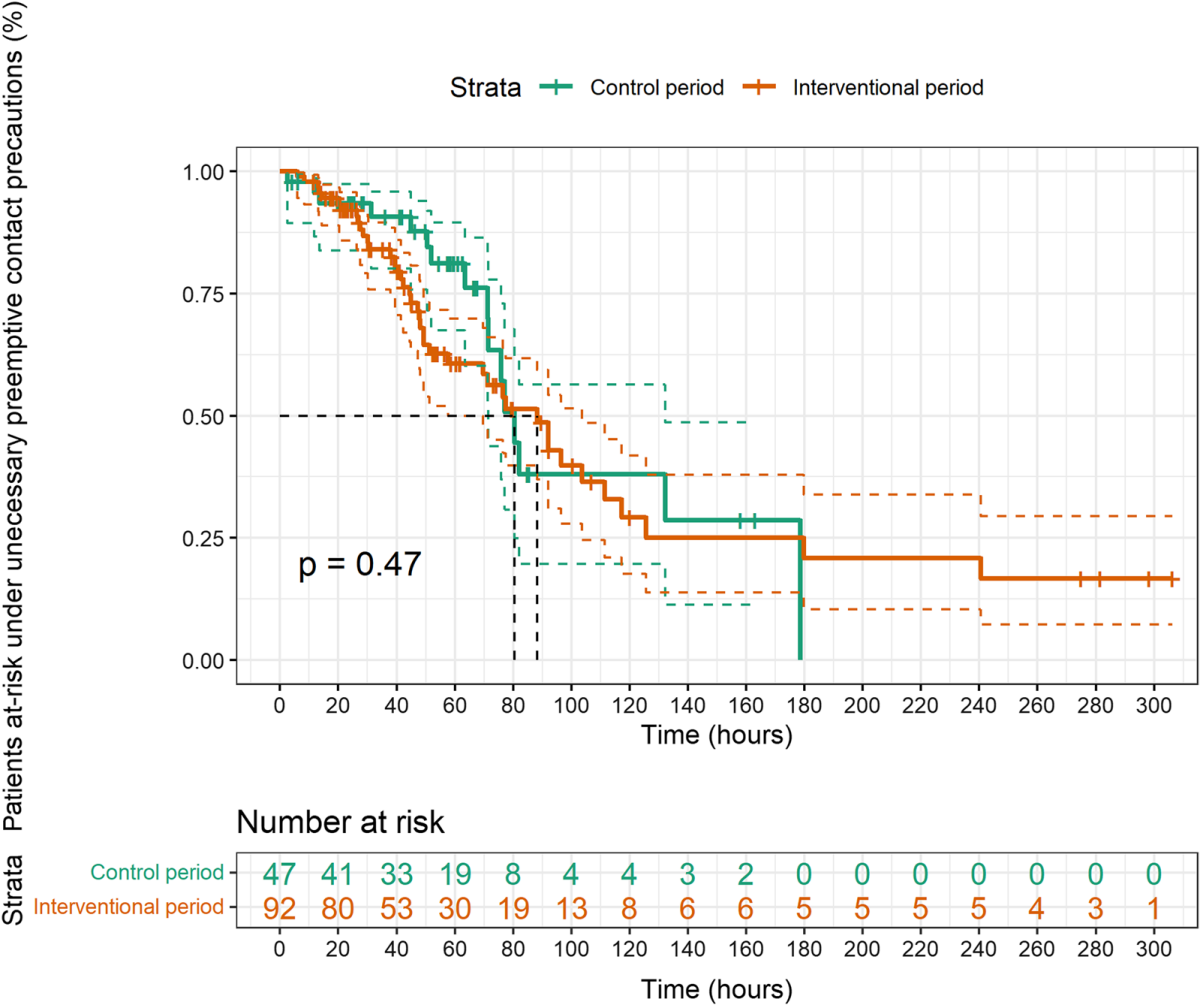
Time (hours) spent under preemptive contact precautions by negative patients screened at admission with culture-based methods (control period) and LAMP assay (interventional period)

Of 92 and 47 patients screened at admission with unnecessary preemptive CP during the interventional and control period, we observed a median time from admission to CP discontinuation of 88.3 (95% CI 57.7–103.7) versus 80.5 (95% CI 71.5–132.1) hours (*p* = 0.47, Fig. 2). After taking into account censoring by discharge, time from admission to result notification was, respectively, 20.8 (95% CI 18.5–25.1) and 64.9 (95% CI 59.8–86.6) hours (*p* < 0.001). We observed that TATs remained stable throughout the study period (Additional file 1: Figure S3). Following univariate competing risk regression, the rapid screening strategy did not accelerate discontinuation of CP (sHR 1.4 [95% CI 0.8–2.6], *p* = 0.2), with similar estimates using Cox regression. Results were unchanged after the exclusion of patients screened during weekends and laboratory holidays, as well as after the exclusion of patients at a high risk (Additional file 1: Appendix S4, Figures S4 and S5).

Among patients admitted during weekdays and screened at admission in the interventional and control period, 34 (37.0%) and 14 (29.8%) had CP discontinued in the ICU. Among them, CP discontinuation occurred 43.4 (IQR 27.0–92.0) and 67.4 (34.7–84.6) hours after admission (*p* = 0.29, Table 3) during the interventional and control period, respectively, and results were notified 22.1 (IQR 12.3–55.2) and 61.9 (56.7–105.0) hours after admission (*p* < 0.001). No apparent impact of the first pandemic wave was observed on pre-analytical and post-analytical TATs (data not shown).

**Table 3:** Laboratory turn-around times among patients at-risk with unnecessary contact precautions and actionable results (excluding patients at a high risk of carriage and patients screened during holidays)

	Interventional period (*n* = 34)	Control period (*n* = 14)	*p* value
*Pre-analytical TAT*			
From admission to screening (h)	11.6 (IQR 2.0–21.8)	6.2 (IQR 3.2–33.3)	0.759
*Analytical TAT*			
From screening to arrival in the laboratory (h)	2.5 (IQR 1.5–11.3)	6.4 (IQR 2.3–19.0)	0.189
From receipt to result notification (h)^a^	2.6 (IQR 2.1–28.8)	40.4 (IQR 29.3–73.7)	< 0.001
*Post-analytical TAT*			
From result notification to CP discontinuation (h)^a,b^	24.0 (IQR 5.7–32.8)	17.4 (IQR 9.1–30.5)	0.56
*Total TAT*			
From admission to CP discontinuation (h)	43.4 (IQR 27.0–92.0)	67.4 (IQR 34.7–84.6)	0.29
From admission to result notification (h)^a^	22.1 (IQR 12.3–55.2)	61.9 (IQR 56.7–105.0)	< 0.001

^a^Excluding 2 patients in the interventional period with missing date of results

^b^Excluding 3 and 5 patients in the interventional and control period with CP discontinued before results notification

CP were implemented for two patients newly CPO-positive by LAMP. However, they were false positive by culture, leading to unnecessary contact precautions. Considering the poor PPV of this test, ICU physicians decided to stop using the LAMP assay on rectal swabs in June 2019.

Incidence densities of acquisition per 1000 patient-days during the interventional and control period were, respectively, 2.48 [95% CI 1.55–3.75] and 2.92 [95% CI 1.46–5.22] for nEcESBL-PE; 0.34 [95% CI 0.07–1.00] and 2.12 [95% CI 0.92–4.18] for CPO; and 0.11 [95% CI 0.03–0.81] and 1.06 [95% CI 0.29–2.72] for CPE only. Incidence rate ratios for ICU-acquired nEcESBL-PE and CPO were 0.80 [95% CI 0.36–1.75; *p* = 0.57) and 0.23 [95% CI 0.03–1.76; *p* = 0.16), after adjustment for colonization pressure and hand hygiene compliance.

## Discussion

The findings of this interventional cohort study support three main conclusions: (1) The diagnostic accuracy of the LAMP assay performed directly on rectal swabs was suboptimal; (2) under real-life conditions, there was no benefit of this rapid diagnostic strategy in a low-endemicity setting, neither for discontinuing unnecessary CP among critically ill patients screened at admission, nor for implementing CP among newly positive patients; and (3) many ICU patients screened at admission and placed under preemptive CP were negative, and most of them were discharged before discontinuation of CP.

The rapid screening strategy had methodological flaws. Although it demonstrated acceptable NPV for discarding intestinal carriage of nEcESBL-PE and CPO, it generated several false positive results as compared to cultures. The low endemicity and poor pre-test probability during universal weekly screening both impacted the observed PPV. Because of the human and economic cost of unnecessary CP, ICU physicians decided to stop using the rapid screening strategy to implement CP early in the study. Moreover, the investigation of discordant results observed that when retesting isolates from previously LAMP-negative swabs, they were often identified as positive. Retesting isolates probably improved sensitivity compared to rectal swabs, due to increased bacterial load and decreased potential Bst DNA polymerase inhibitors (fecal material). However, this problem results from the sampling source rather than sampling quality. Unfortunately, few samples were available to re-examine swabs positive by LAMP but negative by cultures. We hypothesized these discordant results might partly be explained by under-detection from cultures, in case of low bacterial load, non-viable species on specimen, growth difficulty in selective media for non-Enterobacterales species (e.g., non-fermentative bacteria), enzyme mutants not expressing carbapenemases activity [20], and for certain resistance mechanisms with low hydrolytic activity (OXA-48-like enzymes), which has also been observed in prior studies [21, 22], with unclear infection control relevance [22].

Despite reduced analytical TAT, the overall duration of unnecessary CP among patients screened at admission was not significantly different between the intervention and control periods, even after exclusion of patients screened during holidays or at a high risk of carriage. Non-significant reduction of overall TAT when using conventional culture-based methods despite longer analytical TATs is potentially explained by shorter pre- or post-analytical TATs, suggesting a role for external factors. [23] These factors, and shorter ICU length of stay, might also explain the fraction of eligible patients screened at admission with CP discontinuation before ICU discharge. Factors influencing post-analytical TAT might include the absence of computerized rapid reporting pathways regarding result notification, increased workload or under-staffing of both ICU and IPC nurses, and finally the need of IPC consultations, sometimes required to estimate the risk and guarantee adequate interpretation of test results. Direct communication with IPC teams or frontline physicians could accelerate post-analytical TATs. However, the observed poor diagnostic performance limits practical implementation of results for infection control purposes or guiding empiric antibiotic treatment decisions.

We observed a non-statistically significant increase in CPO acquisition rates during the control period, but not regarding nEcESBL-PE. This increase was confounded by an outbreak of VIM-producing *P. aeruginosa* from April 2018 to September 2020 related to an environmental reservoir, with a peak observed in August 2020 [15]. However, similar conclusions were observed regarding acquisition rates after exclusion of non-fermentative bacteria. We are therefore confident that the rapid screening strategy did not change nEcESBL-PE and CPO acquisition rates.

Several experimental studies observed reliable concordance of the eazyplex® SuperBug CRE system when performed on CPO and nEcESBL-PE isolates when compared to cultures or PCR [24–27]. However, when directly performed on rectal swabs, Yamamoto et al. observed a PPV of 62% to detect carbapenem-resistant *Acinetobacter baumannii*, [28] which is close to our PPV. The sole study evaluating clinical relevance of LAMP when performed on rectal swabs and/or bronchial aspirates observed a decreasing incidence of carbapenem-resistant *A. baumannii* infection from 35.2 to 20.9 per 1000 patient-days in a hyper-endemic ICU using weekly, admission, and discharge screening [28]. Another benefit of such rapid test has been suggested by a study using PCR to accelerate screening during outbreaks [29]. However, one should also consider current limitations to implement LAMP tests in routine screening, which include their cost, and the additional workload to simultaneously process cultures and LAMP tests.

This study is the first to evaluate clinical effectiveness of a rapid screening strategy based on LAMP tests to accelerate discontinuation or implementation of infection control measures. However, our study has several limitations. First, the study design did not allow a concurrent control group, which left room open for confounding events such as the COVID-19 pandemic and VIM-producing *P. aeruginosa* outbreak, with a possible influence on surveillance and implementation of infection control measures. The first COVID-19 pandemic wave in March–April 2020 increased workload and modified screening and infection control policies, incl. universal gloving, which might have had an impact on nEcESBL-PE and CPE rates. However, we observed that TATs remained stable during the overall study period. Second, the microbiological laboratory of our institution automated its plating and incubation processes in March 2019, thereby significantly decreasing TAT of cultures [17]. Comparison of LAMP with a competing, improved control could potentially underestimate its true benefits. However, the major problem remained unchanged, which was the short length of ICU stay as compared to the time for CP discontinuation. Third, results might not be generalizable to hyper-endemic settings.

## Conclusion

In its current form, a rapid rectal screening strategy based on LAMP assays has neither a clear benefit to discontinue unnecessary CP among patients screened at admission nor an added value to accelerate the implementation of CP among newly positive patients in a low-endemic setting. This study suggests the requirement for further adjustments, including IT-based automatic reporting of molecular resistance information combined with IPC stewardship to ensure fast and reliable use of results, and further control to improve the quality of rectal swabs.

## Supplementary Information

**Additional file 1.**: Figure S1.

## Data Availability

The datasets used and/or analyzed during the current study are available from the corresponding author on reasonable request.
