# Supplementary material for: An interventional quasi-experimental study to evaluate the impact of a rapid screening strategy in improving control of nosocomial extended-spectrum beta-lactamase-producing Enterobacterales and carbapenemase-producing organisms in critically ill patients
Source: Crit Care. 2022 Jun 7;26:166. doi: 10.1186/s13054-022-04027-8 (PMC9172611; doi:10.1186/s13054-022-04027-8)
Supplement: Supplementary file 1 — Additional file 1.: Figure S1. [file 13054_2022_4027_MOESM1_ESM.docx]

**Additional file 1 Content**

Romain Martischang, Patrice François, Abdessalam Cherkaoui, Gesuele Renzi, Carolina Fankhauser, Jacques Schrenzel, Jérôme Pugin, Stephan Harbarth. An interventional quasi-experimental study to evaluate the impact of a rapid screening strategy in improving nosocomial Extended-Spectrum-Beta-Lactamase Producing Enterobacterales and Carbapenemases Producing Organisms control in critically ill patients.

**Appendix S1.** Adherence to screening and contact precautions

**Appendix S2.** Risk factors for patients screened at admission

**Appendix S3.** Investigation of results with discordant LAMP and culture results

**Appendix S4.** Time to discontinue contact precautions among patients at-risk screened upon admission, after exclusion of patients at a high risk, and patients screened during weekends and laboratory holidays.

**Figure S1.** Monthly rates of antibiotic consumption in Intensive Care Units for targeting ESBL-PE and CPE

**Figure S2.** Weekly screening coverage for patients hospitalized on Monday morning from 5am to 8am.

**Figure S3.** Monthly delay (hours) from admission to discontinuation of CP among patients screened at admission not discharged during the study period

**Figure S4.** Time (hours) spend under preemptive contact precautions by negative patients screened at admission with culture-based methods (control period) and LAMP assay (interventional period) excluding patients screened from Friday to Sunday and during laboratory holidays

**Figure S5.** Time (hours) spend under preemptive contact precautions by negative patients screened at admission with culture-based methods (control period) and LAMP assay (interventional period) excluding patients at a high risk and patients screened during laboratory holidays

**Table S1.** Monthly hand hygiene compliance among healthcare workers in ICU

**Table S2.** Prevalence of ESBL-PE and CPO among 3 sub-cohorts of ICU patients, per pathogen and resistance detected by cultures.

This appendix has been provided by the authors to provide readers additional information about this study.

**Appendix S1.** Adherence to screening and contact precautions

Weekly surveillance screenings are often not performed for patients already screened recently (admission screening), and could also be delayed by several days. Therefore, estimating true adherence to weekly surveillance screening should account for prior and delayed screenings. Instead of measuring adherence of screening only on Monday, which would underestimate the true proportion of patients screened, we opted to measure the screening coverage of all ICU patients hospitalized on Monday from 05 am to 08 am (candidates for weekly screening). Among this population, the screening coverage considered those with a screening performed from the prior Tuesday to the next Wednesday. This indicator helps to answer whether carriage status was investigated among patients present at the time of weekly universal screening. During the interventional phase, we investigated missing screenings, and distinguished screening performed elsewhere, performed in ICU but not included in the study, or not performed. Adherence to prescribed CP was assessed by 4 audits spanning the interventional and control period.

**Appendix S2.** Risk factors for patients screened at admission

Of 231 patients with a targeted screening at admission, we distinguished 58 (25%) patients at a high risk requiring sequential screening, and 173 (75%) patients at risk (Table 1). Most frequent high risk exposures included 27 (46%) previously known carriers, 16 (28%) direct transfer from or recent hospitalization in ICUs abroad, and 15 (26%) prior hospitalization in endemic countries. Most frequent exposures defining patients at-risk included 92 (53%) hospital transfer, 18 (10%) prior hospitalization in Swiss or French hospital, 32 (18%) other reasons, and 22 (13%) unknown reason. Among all patients screened upon admission, 185 (80%) had CP implemented at admission. Among them¸46 (25%) had an indication to keep CP, including other MDRO carriage.

**Appendix S3.** Investigation of results with discordant LAMP and culture results

**LAMP positive and culture negative.**

Because of quality concerns, only four from 23 samples with positive LAMP results and negative culture results were further investigated. 14 of 23 results were discordant for CPO results (6 KPC, 3 OXA-181, 3 NDM, 1 OXA-48, 1 KPC & NDM), and 12 of 23 were discordant for ESBL results (9 CTX-M-1, 3 CTX-M-9). Two samples with discordant ESBL results, and two samples with both discordant ESBL and CPO results were retested using PCR, which did not confirm initial results, and detected TEM genes.

**LAMP negative and culture positive.**

27 of 31 isolates negative for ESBL by LAMP, including 21 *E.coli*, 9 *Klebsiella,* 1 *Citrobacter*, 1 *Enterobacter*, and 1 *Pseudomonas* species*,* were further investigated. Sixteen isolates were LAMP positive (11 CTX-M-1, 4 CTX-M-9, 1 for both CTX-M-1 & CTX-M-9), 4 additional isolates were confirmed as non ESBL-PE by disk diffusion methods, and among the 6 isolates tested by PCR, 2 were positive for TEM and SHV, 2 were positive for SHV, and 2 were negative.

**Appendix S4.** Time to discontinue contact precautions among patients at-risk screened upon admission, after exclusion of patients at a high risk, and patients screened during weekends and laboratory holidays.

Results were unchanged after the exclusion of patients screened during weekends and laboratory holidays, (n=95, 77.4 [95%CI 48.1-117.2] and 80.5 [95%CI 63.5-132.1] hours for interventional and control period, p=0.43), as well as after the exclusion of patients at a high risk (n=96, 51.3 [95%CI 44.8-88.3] and 75.9 [95%CI 71.2-82.1] hours for interventional and control period, p=0.06, Suppl. Figures 2 & 3).

**Figure S1.** Monthly rates of antibiotic consumption in Intensive Care Units for targeting ESBL-PE and CPE


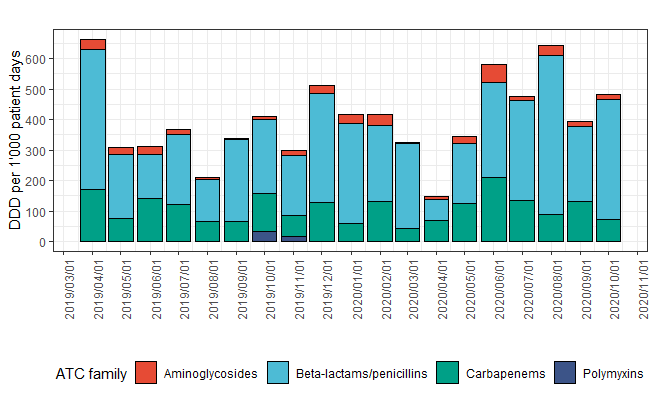


**Figure S2.** Weekly screening coverage for patients hospitalized on Monday mornings from 5am to 8am.


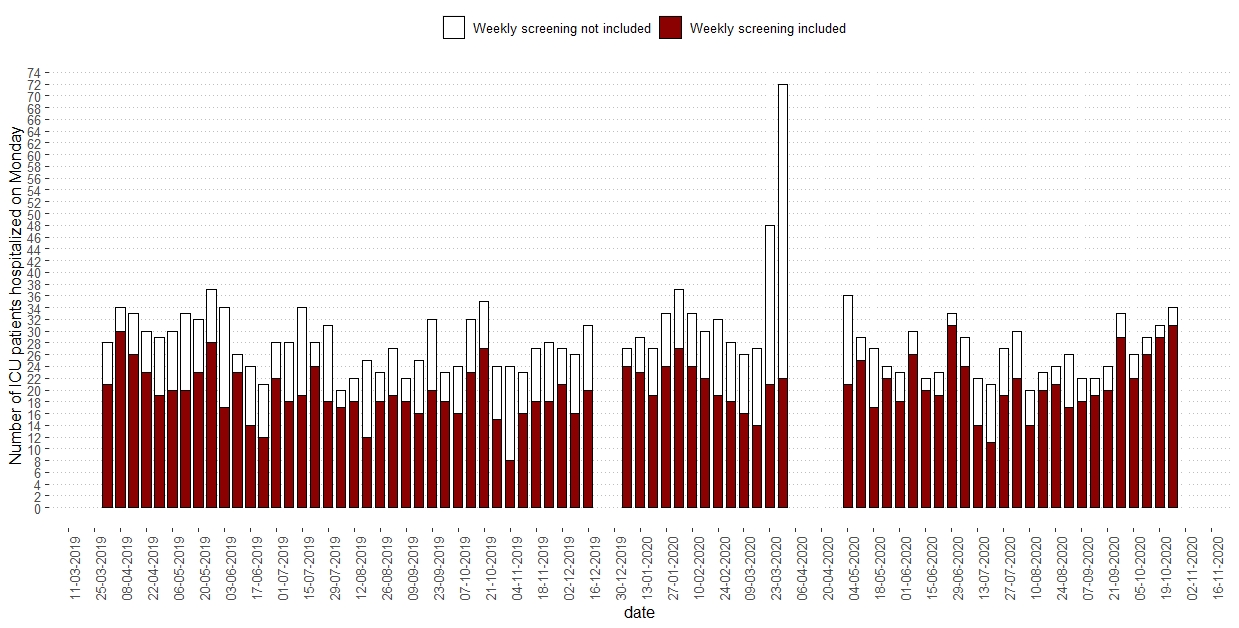


**Figure S3.** Monthly delay (hours) from admission to discontinuation of CP among patients screened at admission not discharged during the study period


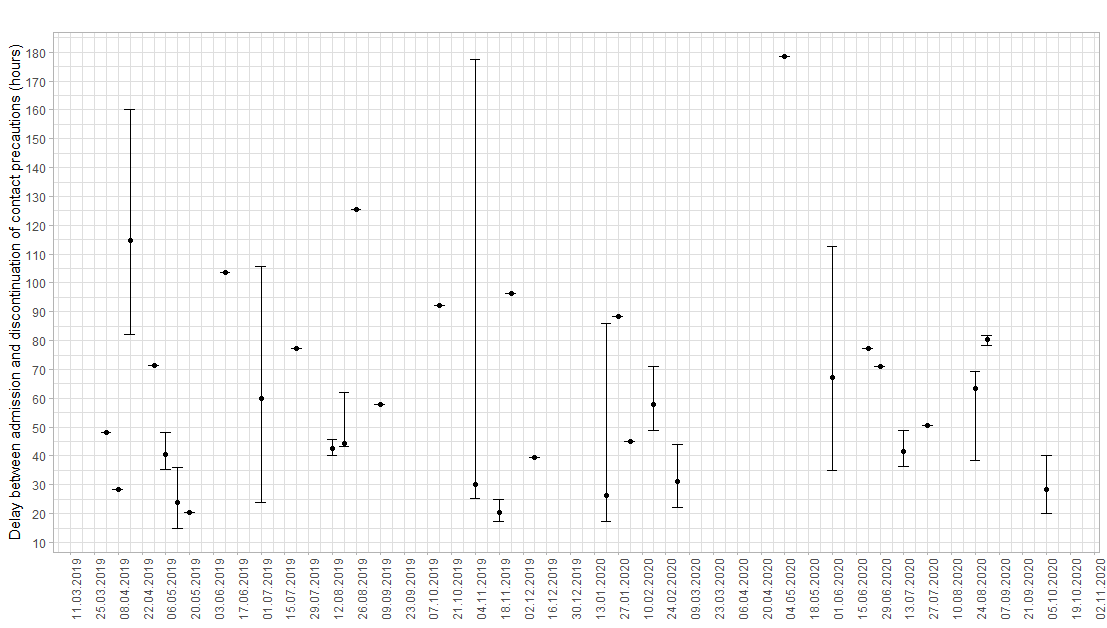


**Figure S4.** Time (hours) spend under preemptive contact precautions by negative patients screened at admission with culture-based methods (control period) and LAMP assay (interventional period) excluding patients screened from Friday to Sunday and during laboratory holidays


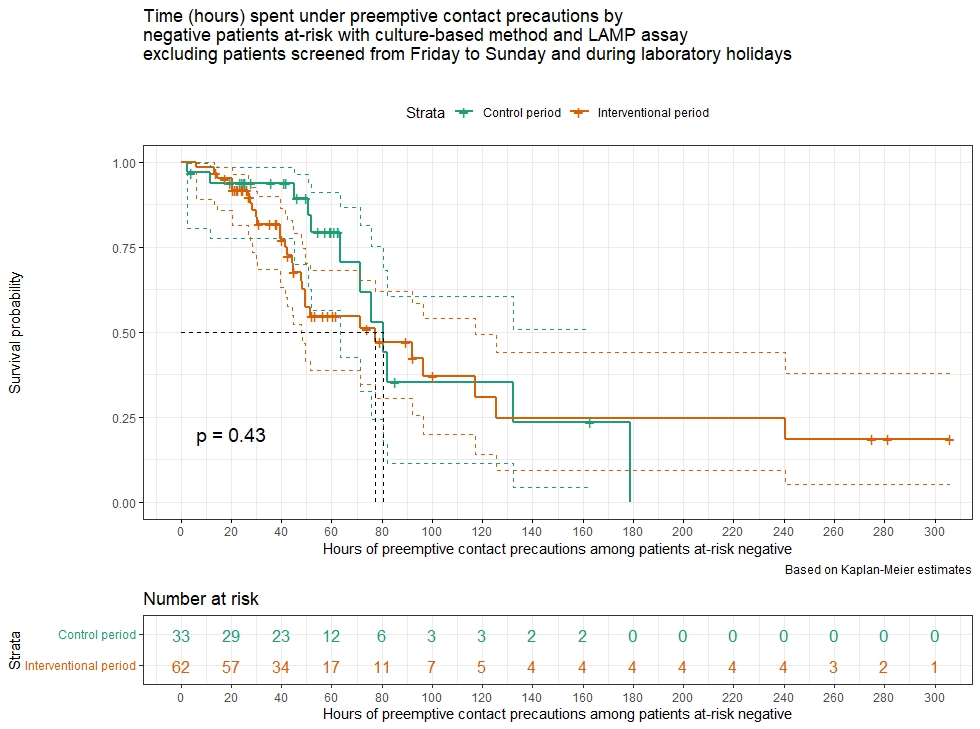


**Figure S5.** Time (hours) spend under preemptive contact precautions by negative patients screened at admission with culture-based methods (control period) and LAMP assay (interventional period) excluding patients at a high risk and patients screened during laboratory holidays


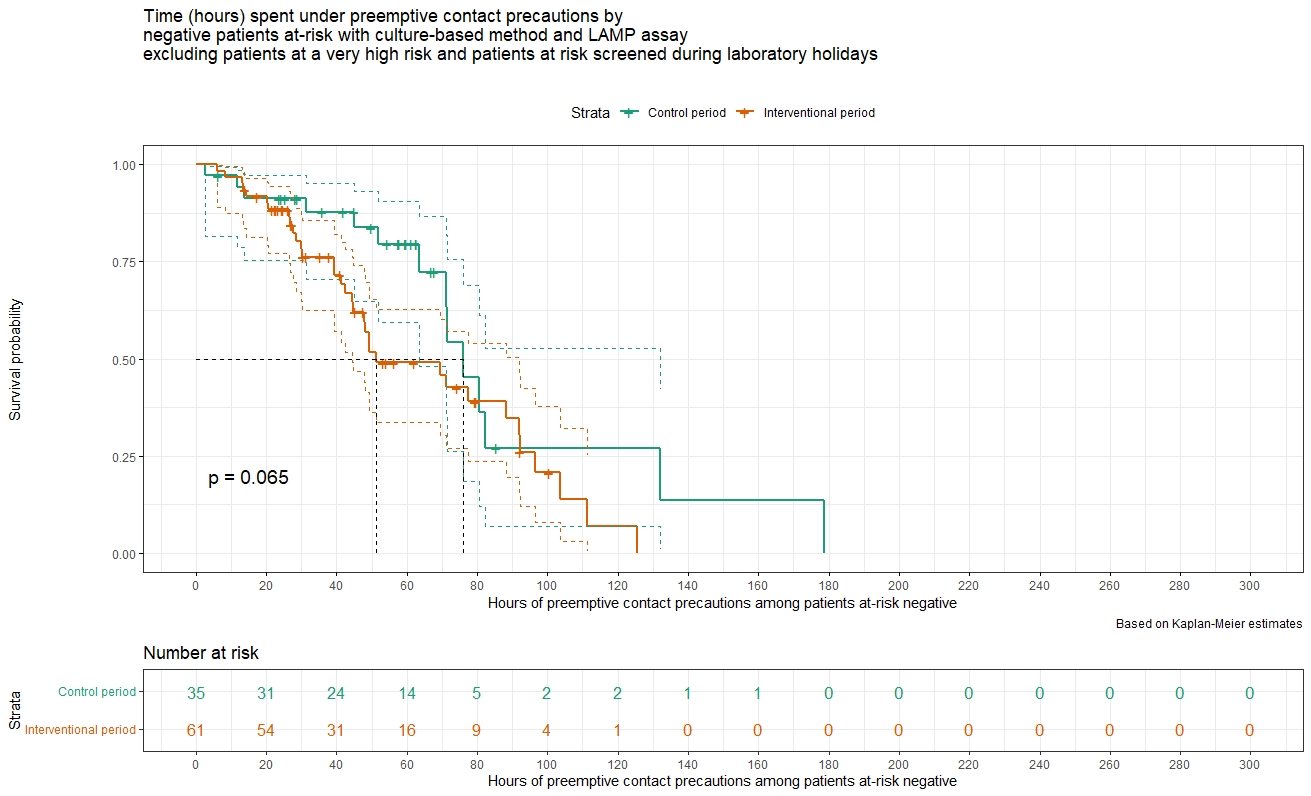


**Table S1.** Monthly hand hygiene compliance among healthcare workers in ICU

| **Date** | **Actions** | **Opportunities** | **Compliance** | **95% CI** |
| --- | --- | --- | --- | --- |
| 2019-05-01 | 31 | 55 | 56,4 | [95%CI 43.3-69.5] |
| 2019-06-01 | 31 | 60 | 51,67 | [95%CI 39-64.3] |
| 2019-08-01 | 17 | 26 | 65,4 | [95%CI 47.1-83.7] |
| 2019-09-01 | 47 | 76 | 61,8 | [95%CI 50.9-72.8] |
| 2019-10-01 | 53 | 79 | 67,1 | [95%CI 56.7-77.5] |
| 2019-11-01 | 30 | 47 | 63,8 | [95%CI 50.1-77.6] |
| 2019-12-01 | 25 | 46 | 54,3 | [95%CI 40-68.7] |
| 2020-01-01 | 18 | 28 | 64,3 | [95%CI 46.5-82] |
| 2020-02-01 | 27 | 47 | 57,4 | [95%CI 43.3-71.6] |
| 2020-03-01 | 4 | 10 | 40.0 | [95%CI 9.6-70.4] |
| 2020-04-01 | 15 | 28 | 53,6 | [95%CI 35.1-72] |
| 2020-05-01 | 17 | 42 | 40,5 | [95%CI 25.6-55.3] |
| 2020-06-01 | 23 | 31 | 74,2 | [95%CI 58.8-89.6] |
| 2020-07-01 | 63 | 110 | 57,3 | [95%CI 48-66.5] |
| 2020-08-01 | 78 | 117 | 66,7 | [95%CI 58.1-75.2] |
| 2020-09-01 | 39 | 60 | 65 | [95%CI 52.9-77.1] |

**Table S2.** Prevalence of patients carrying EC-ESBL, nEC-ESBLPE, CPE, and CPO among 3 sub-cohorts of ICU patients.

|  | **Intervention period** | | | **Control period** | | |
| --- | --- | --- | --- | --- | --- | --- |
|  | Patients at-risk at admission  (n=117) ^a^ | Patients at a high risk at admission (n=30)^b^ | Patients screened weekly (n=589)^c^ | Patients at-risk at admission  (n=57) ^a^ | Patients at a high risk at admission (n=29)^b^ | Patients screened weekly (n=313)^c^ |
| **Patient carrying ESBL-producing *E.coli*** | 15 (12.8%) | 8 (26.7%)^e^ | 81 (13.8%) | 7 (12.3%) | 9 (31.0%) | 36 (11.5%) |
| **Patient carrying nECESBL-PE** | 5 (4.3%) | 5 (16.7%) ^e^ | 19 (3.2%) | 0 (0.0%) | 8 (27.6%) | 16 (5.1%) |
| **Patient carrying CPE** | 0 (0.0%) | 1 (3.3%) | 3 (0.5%) | 0 (0.0%) | 3 (10.3%) | 3 (1.0%) |
| **Patient carrying CPO** | 0 (0.0%) | 1 (3.3%) | 4 (0.7%) | 0 (0.0%) | 3 (10.3%) | 8 (2.6%) |

Footnote

^a^: 1 patients was included both in the interventional and control phase.

^b^: 1 patients were included both in the interventional and control phase.

^c^: 6 patients were included both in the interventional and control phase, patients at-risk were included if screened weekly
